# Tool-Use Model to Reproduce the Goal Situations Considering Relationship Among Tools, Objects, Actions and Effects Using Multimodal Deep Neural Networks

**DOI:** 10.3389/frobt.2021.748716

**Published:** 2021-09-28

**Authors:** Namiko Saito, Tetsuya Ogata, Hiroki Mori, Shingo Murata, Shigeki Sugano

**Affiliations:** ^1^ Department of Modern Mechanical Engineering, Waseda University, Tokyo, Japan; ^2^ Department of Intermedia Art and Science, Waseda University, Tokyo, Japan; ^3^ National Institute of Advanced Industrial Science and Technology (AIST), Tokyo, Japan; ^4^ Future Robotics Organization, Waseda University, Tokyo, Japan; ^5^ Department of Electronics and Electrical Engineering, Keio University, Kanagawa, Japan

**Keywords:** tool-use, manipulation, multimodal learning, recurrent neural networks, deep neural networks

## Abstract

We propose a tool-use model that enables a robot to act toward a provided goal. It is important to consider features of the four factors; tools, objects actions, and effects at the same time because they are related to each other and one factor can influence the others. The tool-use model is constructed with deep neural networks (DNNs) using multimodal sensorimotor data; image, force, and joint angle information. To allow the robot to learn tool-use, we collect training data by controlling the robot to perform various object operations using several tools with multiple actions that leads different effects. Then the tool-use model is thereby trained and learns sensorimotor coordination and acquires relationships among tools, objects, actions and effects in its latent space. We can give the robot a task goal by providing an image showing the target placement and orientation of the object. Using the goal image with the tool-use model, the robot detects the features of tools and objects, and determines how to act to reproduce the target effects automatically. Then the robot generates actions adjusting to the real time situations even though the tools and objects are unknown and more complicated than trained ones.

## 1 Introduction

### 1.1 Background

Tool-use is critical for realizing robots that can accomplish various tasks in complex surroundings. By using tools, humans are capable of compensating for missing bodily functions, greatly expanding the range of tasks they can perform Gibson and Ingold (1993) and Osiurak et al. (2010). By using tools, robots can overcome physical limitations and perform complex tasks. They could also adapt to environments without changing or adding actuators or other mechanisms, reducing their weight and size and making them safer, less expensive, and easier to be used in the real world. Robots capable of working alongside humans and performing daily tasks are increasingly becoming a key research topic in the robotics field Yamazaki et al. (2012) and Cavallo et al. (2014). Tool-use by robots would result in introducing robots into everyday spaces to assist human lives.

Much research has aimed at realizing robots that can perform tool-use tasks, most using preset environments and pre-made numerical models for the target tools and objects. Following those models, the robots calculate and move along optimal motion trajectories. This approach has realized highly accurate and fast movements, allowing robots to perform pan tosses Pan et al. (2018), make pancakes with cooking tools Beetz et al. (2011), cut bread with a knife Ramirez-Amaro et al. (2015), and serve food with a spatula Nagahama et al. (2013) in past studies. This approach can be efficient and realize high performance if the robots are dedicated to a particular purpose in a specific environment, but as the number of tools and objects or task types increase, it becomes difficult to design numerical models of properties and environments for each condition. In addition, it is difficult to deal with the situations when somethings that did not supposed to happen, because the robots can only move as initially instructed by humans.

There are unlimited conceivable situations in which robots might perform various tasks, so preparing tailored tools and individually teaching robots how to use them is impossible. It is thus critical for robots to acquire “tool-use ability,” and to consider how to use tools to achieve a goal without human assistance, even when tools are seen for the first time. We therefore develop a robot to plan and make an action by itself only by showing the goal of the tasks.

### 1.2 Relationships Among Tools, Objects, Actions and Effects in Tool-Use

To acquire tool-use ability, considering relationships among tools, objects, actions and effects is important. According to Gibson (1977) and J.Gibson (1979), affordance is defined as the possibility of an act presented to an agent by an object or environment. The definition of affordance by Gibson is highly conceptual, so there have been attempts to clarify it Turvey (1992), Norman (2013), Chemero (2003), and Stoffregen (2003). Especialy, Chemero argued that affordance is not unique to the environment and it is a relationship between agent abilities and environmental features, providing agent actions. For example, consider a hammer. The hammer head is suitable for hitting objects with strong blows, and the long handle is suited to grabbing and swinging. Therefore, when dealing with nails, the hammer affords hitting. However, if you do not have a hammer, you could hit a nail with a block having similar features, such as hardness, ruggedness, and weight. The hammer can also be used for other purposes. If you want to pull and obtain something that is out of reach, you can use a hammer to pull it. So a long hammer can also propose agents to pull and provide an effect of pulling objects. In other words, tool-use arises not only from tool features, but also from the operated objects, actions to take and the expected effects (i.e., goal). Acquisition of affordance is one of the most essential topic in the cognitive field Bushnell and Boudreau (1993), Piaget (1952), Rat-Fischer et al. (2012), and Lockman (2000), and has also been discussed in robotics field and has gained significant support Horton et al. (2012), Min et al. (2016), and Jamone et al. (2018). Inspired from this theory, we construct the tool-use model with DNNs simultaneously considering features of tools, objects, actions,and effects. By recognizing the relationships among the four factors, we expect the robot to deal with tool-use tasks including those never experienced before.

### 1.3 Research Objective

Our objective is to develop a robot capable of acquiring relationships of four factors: 1) tools the robot can use, 2) target objects that can be manipulated by those tools, 3) actions performed by the robot, and 4) effects by those actions. And to allow the robot to conduct tool-use actions according to the relationships to reproduce providing goals.

We take the approach of developing a robot from own task conduction experiences and allowing it to acquire the relationships. The robot is developed in the following steps. First, multimodal sensorimotor data are recorded while the robot is controlled to conduct tool-use tasks, like it plays to repeat manipulating objects. Then deep neural networks (DNNs) are trained and used to construct a tool-use model, which uses the recorded dataset to learn sensorimotor coordination and relationships among tools, objects, actions, and effects. Then for tests, the robot generates motions for handling novel tools and objects by detecting their features with the tool-use model, and acts to achieve its goal. We verified that the robot can detect the features, recognize its goals, and act to achieve them.

Some of the tools that infants firstly develop to use is stick- or rake-like tools such as forks or spoons, allowing them to extend their reach to grasp or move distant objects. Similarly, we start by allowing the robot to learn to move objects by such simple tools. Specifically, the robot uses I- or T-shaped tools to extend their reach and pull or push an object to roll, slide, or topple.

The research goal is to enable the robot to perform tool-use operations solely from provided goals and reproduce the situations. The experimenters only present a goal image to the robot. We use an image because it clearly and easily shows the target position and orientation of the object. The robot needs to understand the relationships and detect features of the tool and the object, then generates an action toward the goal effect. To manipulate the object to the target position at the target orientation, the robot must adjust operating movements every time step according to the current situation. The goal cannot be completed just by replay actions as same as the trained ones.

The following summarizes our contributions:

• The tool-use model learns relationships of tools, objects, actions, and effects.
• The robot generates tool-use actions based on the detected relationships and current situations even with unknown tools and objects.
• The robot accomplishes tasks solely from a provided goal image, and reproduce the situations.

## 2 Related Works

In this section, we show some related tool-use works except for which use initially prepared numerical models of actions for fixed target or environments.

### 2.1 Understanding Tool Features

Myers et al. (2015) and Zhu et al. (2015) investigated autonomous understanding of tool features, introducing frameworks for dividing and localizing tool parts from RGB-D camera images. These frameworks identify several tool parts that are critically involved in tool functions and recognize tool features relying on those parts. Robots could thereby understand how tools and their parts can be used. Similarly, we allow robots to recognize tool features from their appearance. Moreover, we not only allow the robot to recognize features but also generate tool-use motions.

### 2.2 Planning Tool-Use Motions With Analytic Models

Stoytchev (2005), Brown and Sammut (2012), and Nabeshima et al. (2007) realized robots capable of performing tool-use motions by constructing analytic models. In particular, Stoytchev (2005) controlled a robot to move a hockey puck with various shaped tools, making a table showing object movements corresponding to the shape and shift direction of tools used during observations. By following this table, the robot could carry the object to a target position. Brown and Sammut (2012) presented an algorithm for discovering tool-use, in which the system first identifies subgoals, then searches for motions matching next subgoals one-by-one. They realized a robot capable of grasping various shaped tools and carrying an object. Nabeshima et al. (2007) constructed a computational model for calculating tool shapes and moments of inertia, developing a robot capable of manipulating tools to pull an object from an invisible shielded area, regardless of the shape of the grasped tool. However, these systems need prepared tables or computational models before using tools, making it difficult to deal with unknown tools. In addition, modeling errors may accumulate during execution, often resulting in fragile systems.

### 2.3 Generating Tool-Use Motions by Learning

Another line of research on tool-use motion generation is learning tool features from tool-use experience, which is the same as our approach. Nishide et al. (2012) allowed a robot to experience sliding a cylindrical object with various shaped tools, training a DNN to estimate trajectories of object movements that change depending on tool shape and how it slid. Takahashi et al. (2017) constructed a DNN model for learning differences in functions depending on tool grasping positions. This allowed a robot to grasp appropriate tool positions and to generate motions to move an object to a goal. Mar et al. (2018) considered the orientation of tools as well. They controlled a robot to push a designated object in several directions with tools of several shapes and orientations, and recorded the shift length of object movements. They detected tool features by a self-organizing map corresponded to lengths of object shifts. They realized a robot capable of selecting directions to push the objects depending on the tool’s shape and orientation. Saito et al. (2018b) focused on tool selection, setting several initial and target positions for objects and allowing the robot to experience moving objects with several tools. They trained a DNN model that enabled the robot to select a tool of proper length and shape, depending on the designated direction and distance to the object. In every of these studies, robots with learning models learn and recognize tool features and generate suitable tool-use motions according to tool features. However, they dealt with one specified target object and thus did not consider relationships between tools and objects. These tool-use situations are thus limited, because if a different object is provided, it would be difficult to operate it. Goncalves et al. (2014) and Dehban et al. (2016) conducted research to enable robots to consider four factors: tools, objects, actions, and effects. Goncalves et al. (2014) constructed Bayesian networks that express relationships among the four factors, predicting the effect when the other three factors are input. However, they aimed to determine features of tools and objects based on categories such as area, length, and circularity, which were set in advance by the experimenters. It was therefore difficult for the robot to autonomously self-acquire features without requiring predefined feature extraction routines, and also difficult to manipulate arbitrary objects with arbitrary tools without human assistance. Dehban et al. (2016) also expressed relationships among the four factors using DNNs, realizing a robot that could predict or select one factor when the other three are given. In other words, the robot could predict an effect or select an action or tool to use. However, the action types were fixed in advance, so the robot could only select and follow the predesignated motions, making it difficult to deal with objects and tools in unknown positions or objects not moving as expected.

To address these problems, we construct a tool-use model that allows the robot to self-acquire the relationships among the four factors. The model is expected to detect the features of provided tools and objects and to generate actions depending on the situations. The present study is an extension of Ref. Saito et al. (2018a). to improve two points. The first improvement is related to goal images. In the previous study, provided goal images showed situations just after task executions, namely the final position of the robot arm, making it easy for the tool-use model to predict what kind of actions should be conducted. In the present study, we make the robot arm return to its initial joint position after task completion. The goal images thus show the arm at the initial position, and there are no hints regarding actions to take from the images except for object position and orientation. Second, we introduce a force sensor to realize task executions even when the object is occluded by the robot arm or tools. In the previous study, they used only image data as sensory input, making it difficult to operate small objects that can be occluded during movement. Many papers have shown that using both vision and force can improve the accuracy of object recognition in both cognitive field and robotics field Fukui and Shimojo (1994), Ernst and Banks (2002), Liu et al. (2017), and Saito et al. (2021). By constructing the tool-use model with multimodal DNNs, we realize the robot to operate much more complex tools and objects than in the past study.

## 3 Tool-Use Model

In this section, we describe the method for constructing the tool-use model with DNNs.

### 3.1 Overview of the Tool-Use Model

#### 3.1.1 Task Conduction With the Tool-Use Model

**FIGURE 1:**
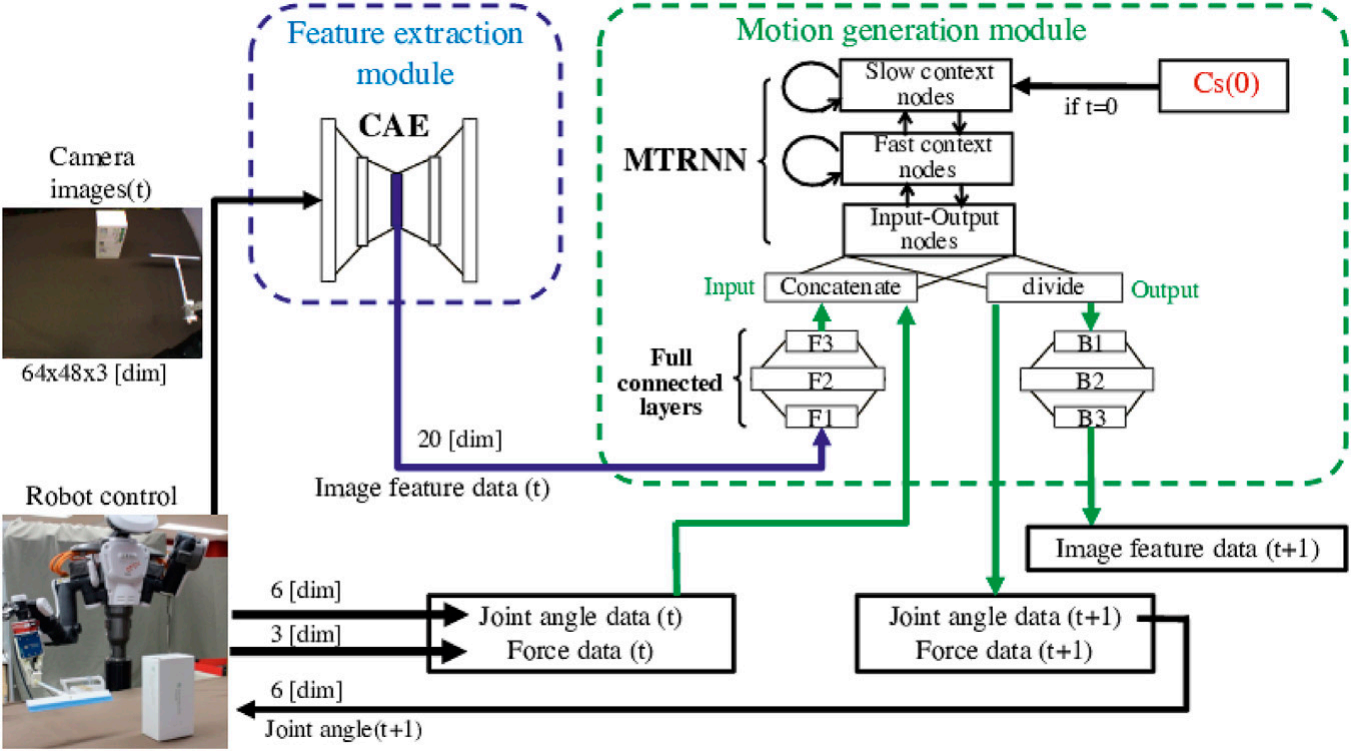
The tool-use model to control the robot to conduct tasks. It comprises a feature extraction module and a motion generation module. In tests, at first, the internal latent space value Cs(0) is explored using a goal image. The Cs(0) detects and expresses features of the tool, object, and actions needed to produce the target effects. For generating a motion, the feature extraction module compresses the number of dimensions in image data and extracts low-dimensional image feature data. Then the motion generation module simultaneously learns image feature data, joint angle data, and force data at the moment, and predicts next-step data. The robot is controlled according to the predicted joint angle data step-by-step.

Figure 1 shows the way to control the robot using the model, which is extended from Ref. Saito et al. (2018a) to deal with both image and force sensor data efficiently. The tool-use model comprises two modules, a feature extraction module and a motion generation module. Since the number of dimensions in image data is considerably larger than in other data, the feature extraction module compresses image data, making the multimodal learning well-balanced at low computational cost. Then, the motion generation module simultaneously learns all time-series data, that is image feature data, joint angle data and force data. This module is expected to learn the relationship among the four factors by using the latent space values Cs(0), which detect features of them from given goal images. It is also expected to generate tool-use actions adjusting in real time by outputting the next joint angle data and move the robot according to the angle data.

#### 3.1.2 Training of the Tool-Use Model

To collect training data, we remotely control a robot to conduct object manipulation tasks with several tools and record the image data, joint angle data, and force data in advance. As for training, in the first step, the image data is used for training the feature extraction module. After that, the motion generation module is trained with time series of the image feature data obtained through the trained feature extraction module, joint angle data and force data.

### 3.2 Feature Extraction Module

We use a convolutional autoencoder (CAE) Masci et al. (2011) to construct the feature extraction module. The CAE is a multilayered neural network with convolutional and fully connected layers, so it has advantages of both a convolutional neural network (CNN) Krizhevsky et al. (2012), which has high performance in image recognition, and an autoencoder (AE) Hinton and Salakhutdinov (2006), which has a bottleneck structure and can reduce data dimensionality. After training, the trained module can represent appearance features such as the shape, size, position, and orientation of tools and objects even when unknown images are input.

Table 1 shows the CAE structure, in which input data pass through the center layer with the fewest nodes, then outputs data with the original number of dimensions. The module is trained so that the output (*y*) restores the input image (*x*), by minimizing the mean squared error (MSE) Bishop (2006) as

$$E=\frac{1}{N}\sum {\left(y-x\right)}^{2}.$$ (1)

Image feature data can be extracted from the center-layer nodes with low dimension. We use a sigmoid function as the activation function for only the center layer, whereas we use the ReLU function for all other layers. The CAE is trained using MSE with the optimizer for the Adaptive Moment Estimation (Adam) algorithm Kingma and Ba (2014).

**TABLE 1:** The CAE structure. The module includes convolution layers and fully connected layers with linear processing. An input data pass through the center layer with the fewest nodes, then outputs data with the original number of dimensions.

Layer	Input	Output	Processing	Kernel size	Stride	Padding
1	(64, 48, 3)	(32, 24, 32)	Convolution	(4,4)	(2,2)	(1,1)
2	(32, 24, 32)	(16, 12, 64)	Convolution	(4,4)	(2,2)	(1,1)
3	(16, 12, 64)	(8, 6, 128)	Convolution	(4,4)	(2,2)	(1,1)
4	(8, 6, 128)	(4, 3, 256)	Convolution	(4,4)	(2,2)	(1,1)
5	3,072	254	Linear	**-**	**-**	**-**
6	254	20	Linear	**-**	**-**	**-**
7	20	254	Linear	**-**	**-**	**-**
8	254	3,072	Linear	**-**	**-**	**-**
9	(4, 3, 256)	(8, 6, 128)	Deconvolution	(4,4)	(2,2)	(1,1)
10	(8, 6, 128)	(16, 12, 64)	Deconvolution	(4,4)	(2,2)	(1,1)
11	(16, 12, 64)	(32, 24, 32)	Deconvolution	(4,4)	(2,2)	(1,1)
12	(32, 24, 32)	(64, 48, 3)	Deconvolution	(4,4)	(2,2)	(1,1)

We tested changing the numbers of the center-layer nodes by 10, 15, 20, 25, and finally set it 20 because it was the smallest number that could sufficiently express the different features of camera images. Therefore, the feature extraction module compresses high-dimensional (9,216 dimensions (64 width × 48 height × 3 channels)) raw image data to 20 dimensions. The module is trained for 5,000 epochs.

### 3.3 Motion Generation Module

For the motion generation module, we use a multiple timescale recurrent neural network (MTRNN) Yamashita and Tani (2008), attaching additional fully connected layers. A MTRNN is a type of recurrent neural network that can predict the next state from a current and previous state. It contains three node types with different time constants: input–output (IO) nodes, fast context (Cf) nodes, and slow context (Cs) nodes. Cf nodes with small time constants learn movement primitives in the data, whereas Cs nodes with large time constants learn sequences. By combining these three node types, long, complex time series data can be learned, the usefulness of which for manipulation has been confirmed in several studies Yang et al. (2016), Takahashi et al. (2017), and Saito et al. (2018b,a, 2020, 2021).

The motion generation module integrates time series of image feature data output from the feature extraction module (*x* ^image^), joint angle data (*x* ^motor^), and force data (*x* ^force^), and predicts next time-step data. Since image data contain more complex and varied information than do other data, we connect fully connected layers before and after IO nodes of only image feature data.

Table 2 shows the structure of the motion generation module, which has settings for the time constants and numbers of each node. We tried to vary numbers of Cs nodes in the range of 8–12, the time constant of Cs nodes in the range of 30–60 in increments of 10, and numbers of Cf nodes in the range of 30–60 in increments of 10. Finally the combination that minimized training error is adopted. If these numbers are too small, complex information cannot be learned, and if they are too large, the module is overtrained and cannot adapt to untrained data. The time constant of Cf had little effect, even when it was changed to around 5. The module is trained for 20,000 epochs.

**TABLE 2:** The structure of the MTRNN attaching fully connected layers. MTRNN contains three node types with different time constants: input–output (IO) nodes, fast context (Cf) nodes, and slow context (Cs) nodes. Since image data contain more complex and varied information than do other data, we connect fully connected layers before and after IO nodes of only image feature data (F1, F2, F3, B3, B2, and B1).

Node name	Number of nodes	Time constant
**F1, B3**	20 (number of image features)	-
**F2, B2**	30	-
**F3, B1**	15	-
**IO nodes**	24	1
**Cf nodes**	50	5
**Cs nodes**	10	40

#### 3.3.1 Forward Calculation

In forward calculations of this module, the internal value is first calculated by fully connected layers (F1, F2, F3) as

$$y_{i}^{image}\left(t\right)=\left\{\begin{array}{cc} tanh\left(\sum_{j\in F1}w_{ij}x^{image}\left(t\right)\right)\; & i\in F2\\ tanh\left(\sum_{j\in F2}w_{ij}y_{j}\left(t\right)\right)\; & i\in F3 \end{array}\right.,$$ (2)

where *x* ^image^(*t*) is the input image feature data, *t* is the time step, *w*_*ij*_ is the weight of the connection between the *j*th and *i*th neuron, and *x*_*j*_(*t*) is the value input to the *i*th neuron by the *j*th neuron.

We concatenate the value, motor data, and force data, then input to the IO nodes of the MTRNN as

$$x_{IO}\left(t\right)=concatenate\left(y_{F3}^{image}\left(t\right),x^{motor}\left(t\right),x^{force}\left(t\right)\right).$$ (3)

We then input the concatenated data to the MTRNN. First, the internal value of the *i*th neuron *u*_*i*_ is calculated as

$$u_{i}\left(t\right)=\left(1-\frac{1}{\tau_{i}}\right)u_{i}\left(t-1\right)+\frac{1}{\tau_{i}}\left[\underset{j\in N}{\sum }w_{ij}x_{j}\left(t\right)\right],$$ (4)

where *N* is the index sets of neural units and *τ*_*i*_ is the time constant of the *i*th neuron (*i* ∈ IO,Cf, Cs). Then the output value is calculated as

$$y_{i}\left(t\right)=tanh\left(u_{i}\left(t\right)\right)$$ (5)

Then the output value *y*_IO_(*t*) is then divided into three parts in charge of image feature data, motor data and force data by their dimensions as

$$y_{IO}\left(t\right)=divide\left(y_{IO}^{image}\left(t\right),y^{motor}\left(t\right),y^{force}\left(t\right)\right).$$ (6)

Then the output value for an image feature data ( $y_{IO}^{image}\left(t\right)$ ) is recalculated with fully connected layers (B1, B2, B3) as

$$y_{i}^{image}\left(t\right)=\left\{\begin{array}{cc} y_{IO}^{image}\left(t\right)\; & i\in B1\\ tanh\left(\sum_{j\in B1}w_{ij}y_{j}^{image}\left(t\right)\right)\; & i\in B2\\ tanh\left(\sum_{j\in B2}w_{ij}y_{j}^{image}\left(t\right)\right)\; & i\in B3. \end{array}\right.$$ (7)

Finally, the predicted image feature data can be obtained as

$$y^{image}\left(t\right)=y_{B3}\left(t\right).$$ (8)

#### 3.3.2 Next-step Data Prediction

The next-step predicted data is calculated as

$$\hat{X}\left(t+1\right)=\left(1-\alpha \right)\times Y\left(t\right)+\alpha \times T\left(t+1\right)$$ (9)

$$\left\{\begin{array}{c} \hat{X}\left(t\right)=concatenate\left({\hat{x}}^{image}\left(t\right),{\hat{x}}^{motor}\left(t\right),{\hat{x}}^{force}\left(t\right)\right)\;\\ Y\left(t\right)=concatenate\left(y^{image}\left(t\right),y^{motor}\left(t\right),y^{force}\left(t\right)\right)\; \end{array}\right.,$$

where 0 ≤ *α* ≤ 1 is the feedback rate and T(*t*) is an input datum, which means training data when we train the module, which in turn means actual data recorded when testing the tool-use model while moving the robot. The predicted value $\hat{X}\left(t+1\right)$ is calculated by multiplying the output of the preceding step *Y*(*t*) and the datum T(*t*) by the feedback rate *α*. The first term presents “closed-loop prediction,” in which the robot associates a data series with past data but not real-time information, which is like a robot simulating inside its head without moving its body. The second term presents “open-loop prediction,” by which the robot repeatedly predicts next-step data from the current situation one-by-one. We can use the feedback rate to adjust predictions. When we train the data, we set feedback rate *α* = 0.1, meaning 90% of input data are previous closed-loop predictions and 10% are recorded training data. When testing a moving robot with actual data, we set the feedback rate *α* = 0.2, meaning 80% of input data are closed-loop predictions and 20% are real-time raw data.

We can control the robot according to predicted joint angle data, namely the value of ${\hat{x}}^{motor}\left(t+1\right)$ . We then input *X* (*t* + 1) data to Eqs. 2, 3 as next input to the motion generation module. In other words, the predicted image feature data and force data ( ${\hat{x}}^{image}\left(t+1\right)$ and ${\hat{x}}^{force}\left(t+1\right)$ ) are not used directly to control the robot but internally used to predict future data with closed-loop predictions. Repeating this process step-by-step, the robot can generate actions.

#### 3.3.3 Backward Calculation

In backward calculation, we use the back propagation through time (BPTT) algorithm Rumelhart and McClelland (1987) to minimize the training error (E), calculated as

$$E=\overset{FinalStep}{\underset{t=1}{\sum }}{\left(Y\left(t-1\right)-T\left(t\right)\right)}^{2}.$$ (10)

We then update the weights as

$$w_{ij}^{n+1}=w_{ij}^{n}-\eta \frac{\partial E}{\partial w_{ij}^{n}},$$ (11)

where *η* is the learning rate, which we set as *η* = 0.001, and *n* is the number of iterations. The initial value of the Cs layer (Cs(0)) is simultaneously updated to store features of the dynamics information as

$${Cs}^{n+1}\left(0\right)={Cs}^{n}\left(0\right)-\eta \frac{\partial E}{\partial {Cs}^{n}\left(0\right)}.$$ (12)

At this time, we start training the module, setting all Cs(0) values to 0. We thus expect that features of tools, objects, actions, and effects will accumulate and self-organize in Cs(0) space, allowing the tool-use model to understand the relationship among the four factors. Therefore, we use the Cs(0) value as latent space. By inputting proper Cs(0) values to the trained network, it is possible to generate actions corresponding to the features of the four factors.

#### 3.3.4 Exploring the Latent Space for Detecting Features From a Goal Image

When the robot deals with unknown tools or objects while testing this tool-use model, the Cs(0) value that best matches the task can be calculated from the trained network, setting error as

$$E={\left(Y\left(0\right)-T\left(1\right)\right)}^{2}+{\left(y^{image}\left(FinalStep-1\right)-T^{image}\left(FinalStep\right)\right)}^{2}$$ (13)

We need to provide initial image, joint angle and force data (T (1)) and final image data (T^image^ (FinalStep)), which is the goal image. At this time, the output value *y* ^image^ (FinalStep −1) is calculated by setting the feedback rate *α* = 0 in Eq. 9, which is fully closed-loop predictions. Then, by altering Cs(0) to minimize error as same as Eq. 12, the model can explore a proper Cs(0) value. This calculation is conducted for 20,000 epochs, by which time it has fully converged.

## 4 Experimental Setup

### 4.1 System Design

We use a humanoid robot, NEXTAGE OPEN developed by Kawada Robotics KAWADA-Robotics (2020) and control it to conduct tasks with its right arm, which has 6 degrees of freedom. The robot has two cameras on its head, which have 9,216 dimensions (64 width × 48 height × 3 channels). We used only its right eye camera. A force sensor developed by WACOH-TECH WACOH-TECH (2020) attached to its wrist records 3-axis force data. A gripper developed by SAKE Robotics SAKE-Robotics (2020) is also attached.

We record joint angle data, an image, and force data every 0.1 s, namely at a sampling frequency of 10 Hz. Before inputting to the tool-use model, force data value are rescaled to [ − 0.8, 0.8], and joint angle data are rescaled to [ − 0.9, 0.9]. Image data are first simply scaled to [0, 255] for the feature extraction module. Then the output image feature data are rescaled to [ − 0.8, 0.8] and input to the motion generation module.

### 4.2 Objects Used in the Experiments

**FIGURE 2:**
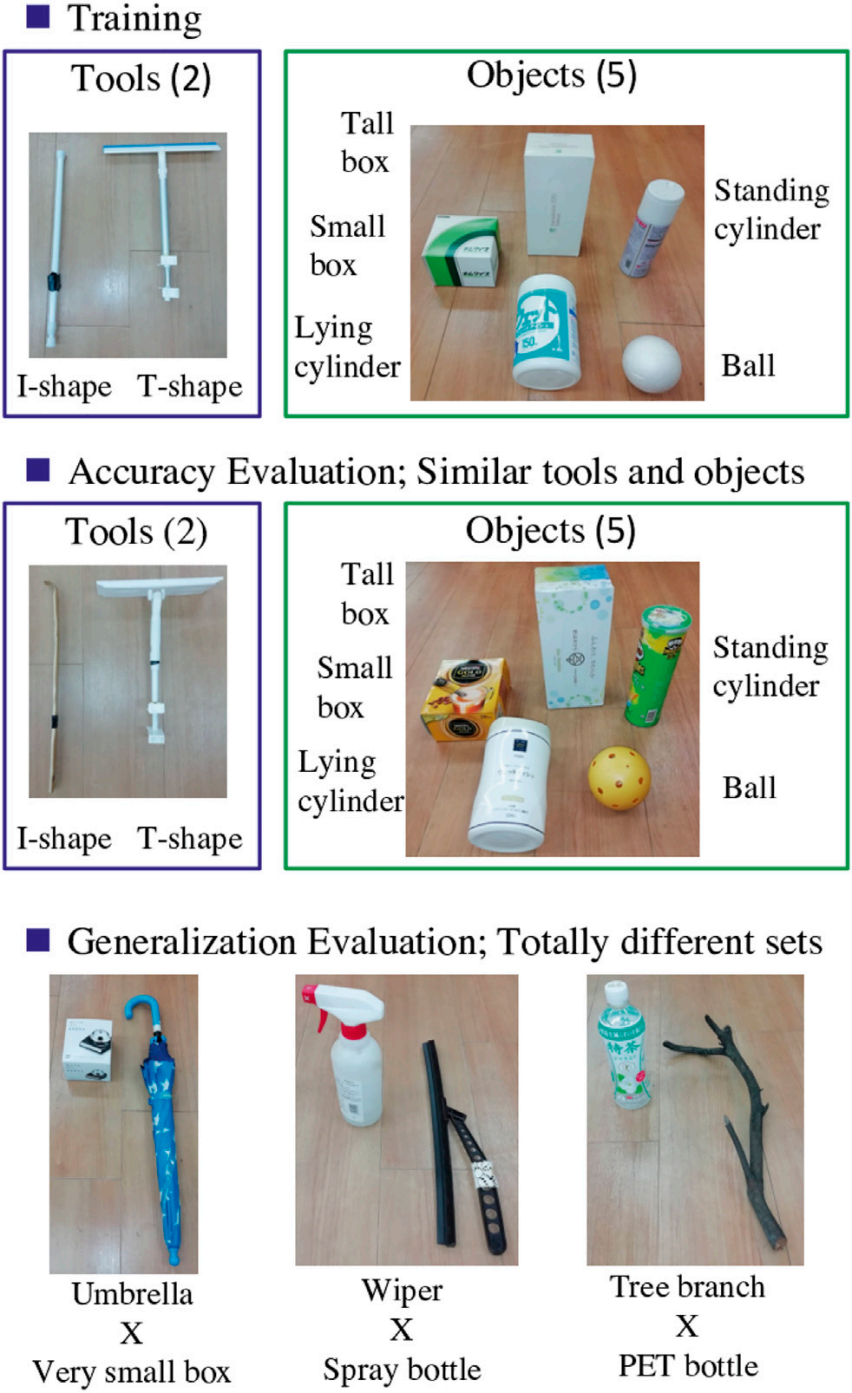
Tools and objects used in the experiments. The upper images show the training setup. The middle images show untrained tools and objects similar to trained ones. These are used to evaluate accuracy of the tool-use model. Images at bottom show untrained tools and objects completely different from trained ones, used to test the model’s generalizability.

**FIGURE 3:**
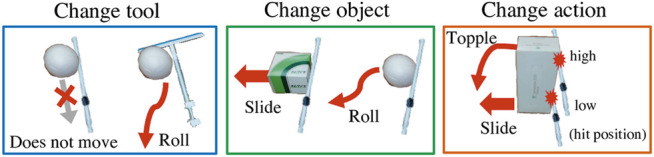
Examples in which effects will differ by tools, objects, and actions. In the left picture, even though the object and the action are the same, the ball will not move if it is pulled with the I-shaped tool, but will roll if pulled with the T-shaped tool. In the middle picture, if we push left with the I-shaped tool, the box will slide but the ball will roll. In the right picture, even when using the same box and same I-shape tool, the effect of object behavior will differ depending on the height at which the robot pushes the box, toppling if pushed at a high point or sliding if pushed at a low point.

Figure 2 shows the tools and objects used in our experiments. For training, we prepare two kinds of tools (I- and T-shaped) and five kinds of objects (ball, small box, tall box, lying cylinder, and standing cylinder), all basic and simple shapes. We choose these to provide a variety of shapes and heights to cause different effects. As the two pictures on the left side of Figure 3 show, tools or objects can change the effects. In the left picture, even though the object and the action are the same, the ball will not move if it is pulled with the I-shaped tool, but will roll if pulled with the T-shaped tool. In the middle picture, if we push left with the I-shaped tool, the box will slide but the ball will roll.

For evaluation experiments, some similar tools and objects, shown in the middle low in Figure 2, are prepared. We also prepared some totally different and more complex tools and objects, such as an umbrella, a wiper, a tree branch, a box much smaller than the trained one, a spray bottle, and a PET bottle. These are shown at the bottom of Figure 2.

### 4.3 Task Design

The robot’s task is to use a tool to move an object to the position and orientation shown in a goal image. The robot must generate actions with the right direction and the right positional height. For example, as on the right in Figure 3, even when using the same box and same I-shape tool, the effect of object behavior will differ depending on the height at which the robot pushes the box, toppling if pushed at a high point or sliding if pushed at a low point. Moreover the robot needs to flexibly adjust the arm angles in real time not just fixing the direction and the height at once. Objects are easy to move with a little force and sometimes behave differently as expected.

We placed one object on a table in front of the robot so that its center position is set in the same position. In all tasks, the robot starts and ends movements at the same home position. We pass the robot a tool before it starts a task, so it initially grips it. This grip is maintained during all movements.

### 4.4 Training Dataset

To record training data, we remotely control the robot with a 3-dimensional mouse controller. For the training data, we designed four kinds of trajectories: sliding sideways or pulling toward the robot, with each action performed at either a high or low position, designed by the remote control. Then the robot is controlled to move according to the trajectories 5 times in each combination of tools and objects. Therefore, there are 200 training datasets (2 tools × 5 objects × 4 actions × 5 trials for each task).

By keeping the robot stationary at its position after task completion until 10.7 s from the start, we record the sensory motor data for 10.7 s in every task, sampling each 0.1 s. There thus are 107 steps for each data.

Table 3 roughly categorizes the effects and summarizes each combination of tools, objects, and actions. There are several effects like “shift to the left,” “shift to the front,” “roll to the left,” “roll to the front,” “topple to the left,” “topple to the front,” and “do not move.” Although we categorized the effects to make them easy to understand, the actual effects differ one by one.

**TABLE 3:** Forty dataset combinations for training whose sensorimotor data is recorded in advance by remote controlling the robot. Effects are roughly categorized and colored differently. We allowed the robot to experience these combinations of tools, objects, actions, and effects for training the tool-use model.

Tool Actions	I-shape				T-shape			
object	Slide to the left low	Slide to the left high	Pull to the front low	Pull to the front high	Slide to the left low	Slide to the left high	Pull to the front low	Pull to the front high
Small box	Shift tothe left	Does not move	Does not move	Does not move	Shift to the left	Does not move	Shift to the front	Does not move
Tall box	Shift to the left	Topple to the left	Does not move	Does not move	Shift to the left	Topple to the left	Shift to the front	Topple to the front
Lying cylinder	Roll to the left	Does not move	Does not move	Does not move	Roll to the left	Does not move	Shift to the front	Does not move
Standing cylinder	Shift to the left	Topple to the left	Does not move	Does not move	Shift to the left	Topple to the left	Shift to the front	Topple to he front
Ball	Roll to the left	Does not move	Does not move	Does not move	Roll to the left	Does not move	Roll to the front	Does not move

There are two differences between “shift” and “roll.” “Shift” is a movement where an object moves beside a tool and stops movement just after the robot stretches its arm to its full extent. In contrast, “roll” is a movement where an object precedes the arm movement and keeps moving for a while after the robot fully extends its arm, so the object moves differently every time and there are a variety of goal positions.

### 4.5 Experimental Evaluation

When testing the tool-use model with actual data taken during moving robot, the setup for recording data and constructing the model is same as in training. However, we set the feedback rate *α* = 0.2 as described in section 3.3.2. Therefore, 80% of input data are closed-loop predictions and 20% are real-time raw data. By increasing the feedback rate from that in training, the robot more easily adjusts to real-time situations.

Three evaluations are conducted:

• analyze the training results,
• evaluate task execution accuracy with similar objects or tools, and
• evaluate generalizability with totally different tools and objects.

#### 4.5.1 Analysis of Training Results

At first, we check if the training can be conducted well. We first confirm that the feature extraction module with the CAE can properly extract image features. We reconstruct the images input by the module and confirm whether the reconstructed images are similar to the input. If the module performed reconstruction well, that means it could accumulate the essential characteristics of images in low-dimensional image feature data.

Second, we evaluate whether the motion generation module with the MTRNN can acquire relationships among tools, objects, actions and effects. We analyze the latent space, the initial step of Cs neuron value (Cs(0)) of each training data trained by Eq. 12 by principal component analysis (PCA). Cs(0) values for similar training data are expected to be clustered and different values should be apart, meaning the Cs(0) space can well express features of the factors of each task. In other words, we can confirm whether the features are self-organized. We analyze three maps of Cs(0) values, each presenting features of tools, objects, and actions.

#### 4.5.2 Accuracy Evaluation

We then conduct evaluation experiments moving the robot. Task execution accuracy is evaluated using unknown objects and tools which are similar to trained ones, shown in the middle of Figure 2. To carefully confirm the robot’s ability to detect both objects and tools, the experiments are conducted with combinations of trained tools and unknown objects, and with unknown tools and trained objects.

We provide the tool-use model with goal images and explore proper Cs(0) value by Eq. 13. The model then generates actions using the explored Cs(0) values, and we confirm whether the robot can reproduce the expected effects. We measure success rates according to object behaviors during robot actions, and the final positions and orientations of the objects. Regarding object behavior, we confirm whether they clearly roll, shift, or topple. Regarding final positions, we regard shift trials as successful if the final object position is within one-thirds distance from the home position to the goal position. Regarding orientation, we regard trials as successful if the difference in inclination between final and target orientations is less than 30°.

We also analyze explored Cs(0) values and check if the tool-use model can detect and express the features of tools, objects and expected actions by comparison with Cs(0) values in the training data. This is confirmed by superimposing PCA results for the explored Cs(0) on the three Cs(0) training data maps described above.

This experiment is conducted by providing 12 goal images with an I-shaped tool, and 16 goal images with a T-shaped tool to show different effects. There are 20 combinations of objects and actions, but there are some same “does not move” effects, as shown in Table. 3. With the T-shaped tool there are also “roll to the left” and “roll to the right” effects that result in random goal positions. Therefore, there are 12 effects for the I-shaped tool and 16 for the T-shaped tool. All tasks are conducted 3 times in both experiments, with combinations of trained tools and unknown objects, and unknown tools and trained objects. Thus the experiment is performed 168 trials ((12 + 16) × 3 × 2).

#### 4.5.3 Generalization Evaluation

In the last evaluation experiment, generalizability of the tool-use model is confirmed. The procedure is same as in Accuracy Evaluation, except that both tools and objects are totally different, as shown at the bottom of Figure 2. We set goal images expecting the robot to slide a very small box in a low position to the left with an umbrella, slide a spray bottle in a high position to topple it with a wiper, and pull a PET bottle in high position to topple it with a tree branch. The experiment is conducted 3 times each, so there are nine trials (3 × 3).

## 5 Result

In this section, we show the result of the training and two evaluation experiments: Accuracy Evaluation and Generalization Evaluation.

### 5.1 Training Analysis

**FIGURE 4:**
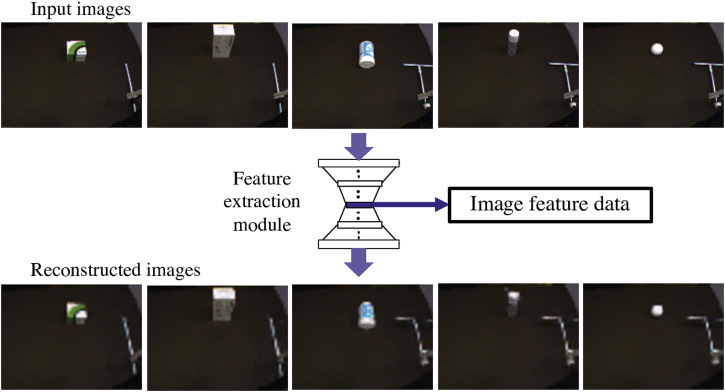
Images reconstruction by the feature extraction module with the CAE. Upper images are original test data with training tools and objects, which are input to the module. The bottom images are reconstructed by the module. All images show their shape and size well, which demonstrates that the feature extraction module can well express image features in output from the center layer.

The reconstructed images in Figure 4 suggest that the trained feature extraction module could reproduce the original input images. The input images shown in the figure are test data not used for training. All are well reconstructed, demonstrating that the feature extraction module could well express image features in output from the center layer.

**FIGURE 5:**
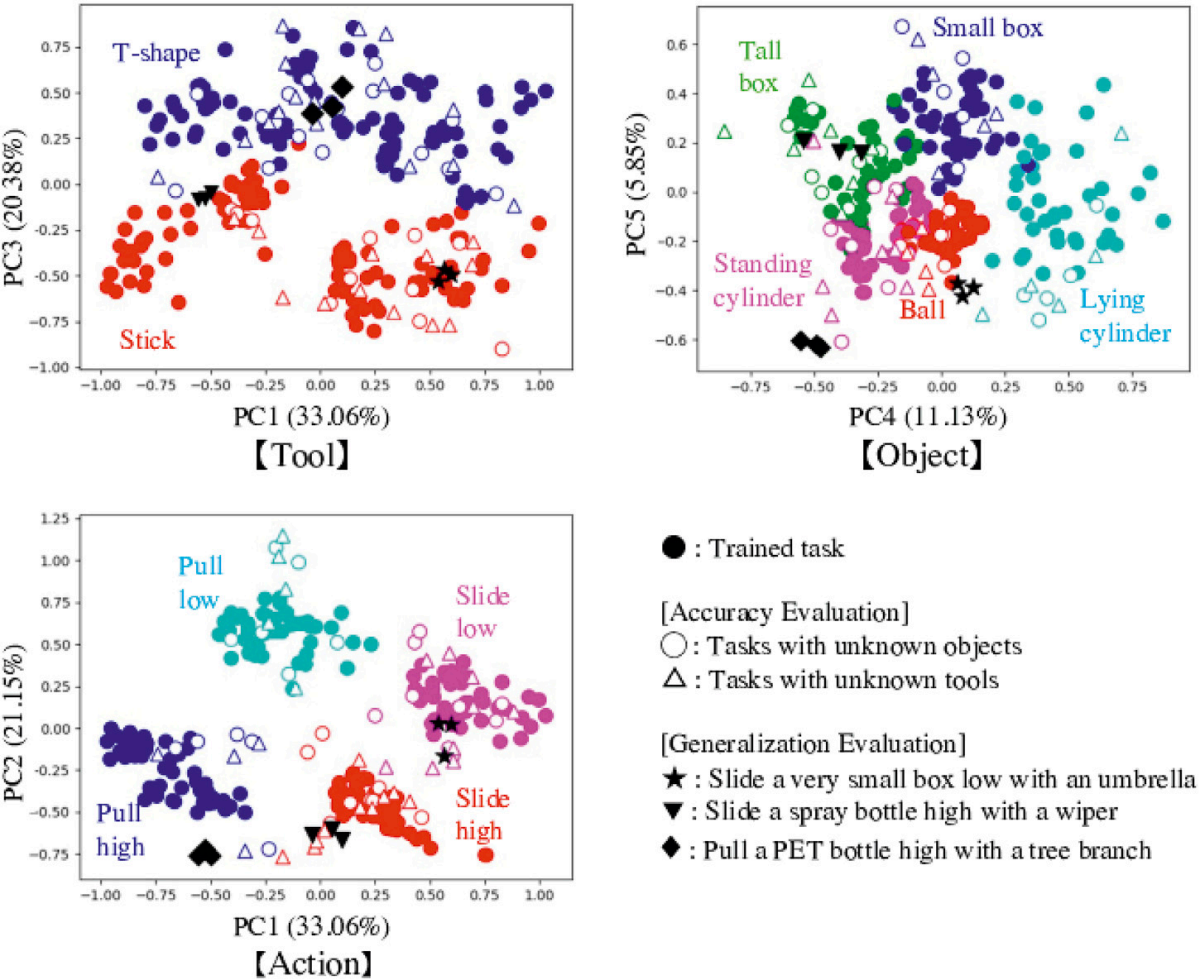
Results of PCA for the internal latent space values, Cs(0). We change the axis to show for expressing map of tools, objects and actions. Trained tasks (solid circles) are separated and clustered according to the features of tools, objects, and actions. Focusing on the map axes, we can also say that PC1 and PC2 represent action features, with PC1 indicating action type (sliding or pulling) and PC2 representing heights. PC3 represents tool features, and PC4 and PC5 represent object features. Cs(0) values could simultaneously express tools, objects, and actions which can reproduce goal effects. Values for explored Cs(0) in the tested tasks are superimposed on the maps as hollow circles and triangles, and black stars, inverted triangles, and diamonds. Many plots are plotted in the proper regions, suggesting good feature detection.

We performed PCA on the internal latent space values, Cs(0) of the training data. The results are shown in Figure 5 as solid circles. The plots are colored according to each feature in the three maps: tools, objects, and actions. As a result, in every map plots of different features are separated and same features are clustered, demonstrating that positions in Cs(0) value maps can express the features. Focusing on the map axes, we can also say that PC1 and PC2 represent action features, with PC1 indicating action type (sliding or pulling) and PC2 representing heights. PC3 represents tool features, and PC4 and PC5 represent object features. Cs(0) values could simultaneously express tools, objects, and actions which can reproduce goal effects.

### 5.2 Accuracy Evaluation

**FIGURE 6:**
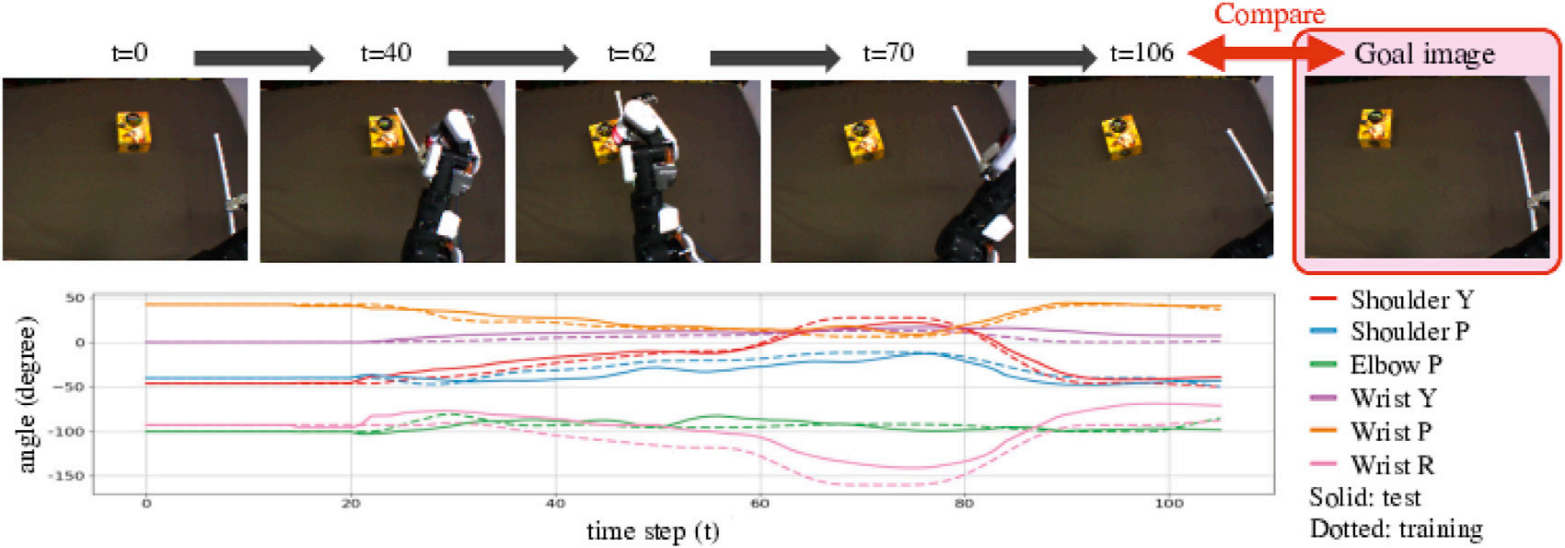
Example result of Accuracy Evaluation using unknown objects and tools shown in the middle of Figure 2. These pictures show camera images while the robot was conducting the task. We provided a goal image showing the untrained small yellow box, shifted to the left from the initial position. The robot then detected features of the tool and the object and predicted a suitable action, generating an action that would properly slide the box in a low position to move it left, reproducing the goal situation. The bottom graph shows the generated angle trajectories with solid lines. We compare the lines to trained angle trajectories for shifting the small box shown in the upper of Figure 2 to the left with dotted lines. The trajectory lines are almost same but sometimes shifted. We can say that the robot could detect proper action type and adjust it depending on the real time situation.

In the experiment of Accuracy Evaluation, the robot is expected to use similar unknown objects or tools. We confirmed whether the robot can reproduce situations in goal images. For example, as Figure 6 shows, the goal image shows the small yellow box shifted to the left. The DNN model explored proper Cs(0) values using the goal images, allowing the robot to generate motions. Figure 6 shows camera images while the robot is moving. In this example, the robot moved properly to make the final image similar to the goal image, matching the success definition rule. We also show the generated trajectory with dotted lines. The lines are similar to but sometimes shifted from the solid lines, that is training trajectory for shifting the trained small box to the left. Therefore, we can say that the robot could detect proper action type and adjust it depending on the real time situation.

Table 4 summarizes success rates. The robot succeeded in performing 83% (70/84) of tasks with unknown objects and 79% (66/84) of tasks with unknown tools. Notably, the robot had more difficulty dealing with tall objects and T-shaped tools. We assume that the reason for this is that they contain topple effects. If we look only at tasks for that effect, the success rate is further reduced to 61% (22/36). The topple effect causes sudden changes in image and force sensor data, causing large sudden changes in DNN model input, making this much more difficult to learn than other effects.

**TABLE 4:** Success rates for Accuracy Evaluation using unknown objects and tools shown in the middle of Figure 2. We confirm whether the robot can reproduce the expected effects. We measure success rates according to object behaviors during robot actions, and the final positions and orientations of the objects.

Untrained objects or tools	Success rate
**Small box**	15/15 (100%)
**Tall box**	16/21 (76%)
**Toppled cylinder**	13/15 (87%)
**Standing cylinder**	14/21 (67%)
**Ball**	12/12 (100%)
**Total untrained objects**	70/84 (83%)
**I-shaped tool**	31/36 (86%)
**T-shaped tool**	35/48 (73%)
**Total untrained tools**	66/84 (79%)
**Combined total**	136/168 (81%)

We also analyzed the explored Cs(0) values and plotted them on the space of trained ones, as shown in Figure 5. There are three trials of each task, but we plotted only one with successful results. All tasks succeeded at least once. Cs(0) values for tasks using unknown objects are shown as hollow circles, and tasks using unknown tools are shown as hollow triangles. Plots of tools and objects are colored according to their shape features. For actions, the plots are categorized and colored according to the most similar trained actions: pull low, pull high, slide low, or slide high. Almost all plots are properly represented in the same feature spaces, meaning the tool-use model could explore proper Cs(0) values and detect features of tools and objects, and expect suitable actions to conduct in untrained tasks.

When plotting Cs(0) values for failed tasks as a test, there were three types of mistaken distributions. Many were plotted at totally different far spaces, indicating the tool-use model could not detect the features. Some were plotted on the middle of feature clusters especially in the action map. This often occurs for the “does not move” effect, where multiple actions are possible to achieve the target. For example, we allowed the robot to experience “pull high,” “pull low,” and “slide high” as training data for behaviors that can reproduce the “does not move” effect in a combination of the ball and the I-shaped tool. We therefore suspect that the robot could not select one action from among the candidates. When we forcibly moved the robot with the ambiguous Cs(0) value, it sometimes mixed some actions and other times just waved its arm near the initial position. Finally, regarding the third mistaken distribution, Cs(0) values are plotted in mistaken combinations of clusters. For example, when the real combination was “T-shaped tool, standing cylinder, slide high, topple to the left,” the model detected this relationship as the combination “T-shaped tool, standing cylinder, pull low, topple forward.” This happened because the robot correctly understood the relationships among the four factors, but misunderstood the effects.

### 5.3 Generalization Evaluation

**FIGURE 7:**
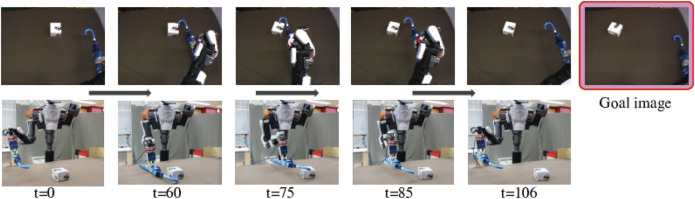
Result of Generalization Evaluation with a very small box and an umbrella shown in the bottom of Figure 2. The pictures are the robot’s eye camera images and the whole view taken by an external camera. We provided a goal image showing the very small box shifted left. The robot could properly detect features and generate a motion to slide to the left, reproducing the situation in the goal image.

**FIGURE 8:**
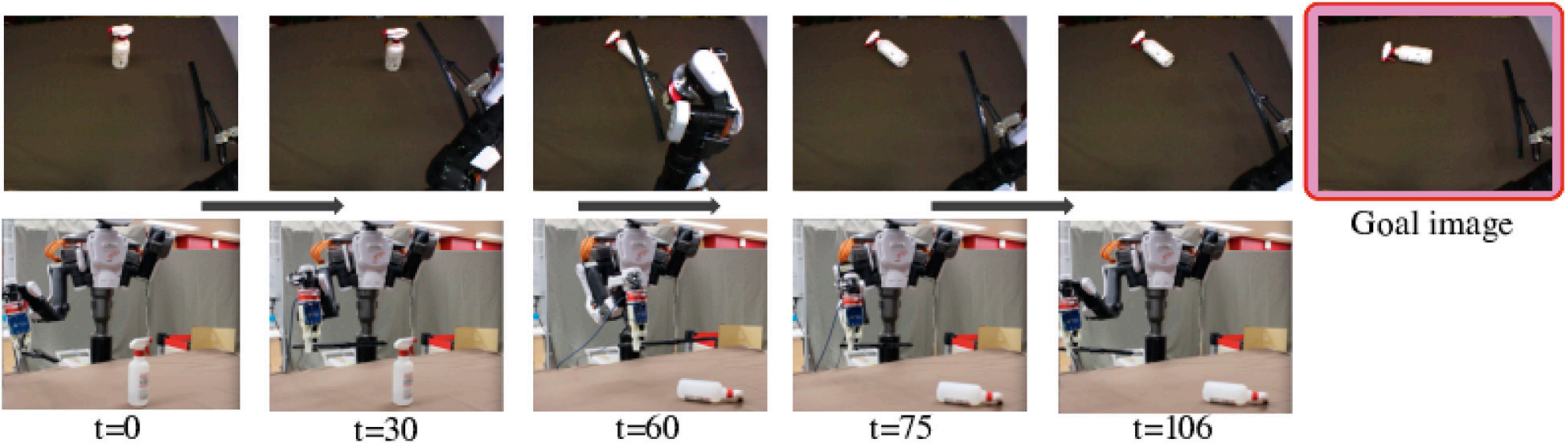
Result of Generalization Evaluation with a spray bottle and a wiper shown in the bottom of Figure 2. We provided a goal image showing the spray bottle toppled to the left. The robot could properly detect features and generate motions to push to the left from a high point and reproduce the situation in the goal image.

**FIGURE 9:**
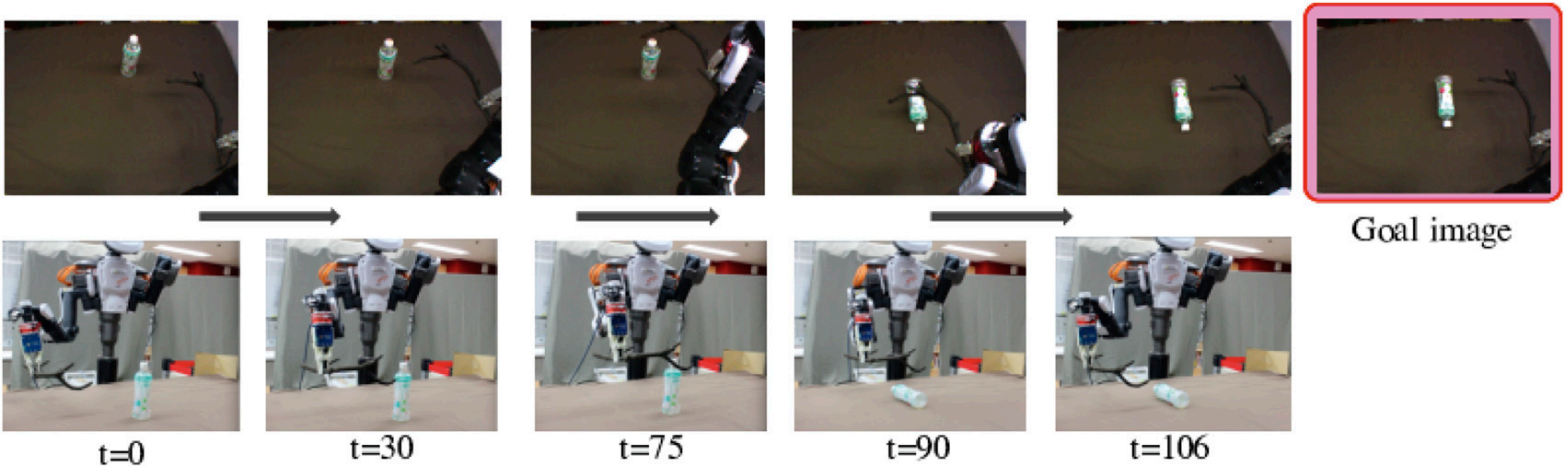
Result of Generalization Evaluation with a PET bottle and a tree branch shown in the bottom of Figure 2. We provided a goal image showing the PET bottle toppled forward. The robot could properly detect features and generate motions to pull forward from a high position, reproducing the situation in the goal image.

In Generalization Evaluation, we used totally different tools and objects. These tools and objects are more complex than trained ones, and thus the objects behave differently from trained situations. Therefore the task cannot be completed just by replay the training actions and the robot needs to adjust its movement step-by-step to manipulate the object with the tool. Figures 7–9 show the results of the robot’s action generation. In the first task, the robot slid the umbrella to the left from a low position and shifted the very small box to the left. In the second task, the robot slid the wiper to the left from a high position and toppled the spray bottle to the left. In the third task, the robot pulled the tree branch to the front from a high position and toppled the PET bottle forward. In all the case, the robot could properly move and reproduce the goal situations, adjusting to the features of tools and objects, and the real time situations. We confirmed tool-use ability of the robot with the tool-use model that can be generalized to use unknown tools and objects.

Figure 5 shows the explored Cs(0) value plotted on the space of trained ones. Black stars show the combination of the very small box and the umbrella, black inverted triangles show the combination of the spray bottle and the wiper, and black diamonds show the combination of the PET bottle and the tree branch. The results suggest recognition by the robot, which had to detect each combination as “tool close to I-shape, ball like object, slide to the left from a low position” in the first task, “tool close to I-shape, tall box like object, slide to the left from a high position” in the second task, and “tool close to T-shape, standing cylinder like object, pull forward from a high position” in the third task. The tools and objects are recognized as ones similar in shape, and actions are also correctly matched. Most importantly, these detected combinations result in the expected effects. These results indicate that the tool-use model could detect the features of tools, objects, actions, and effects considering the four relationships, even if the provided tools and objects are unknown.

**FIGURE 10:**
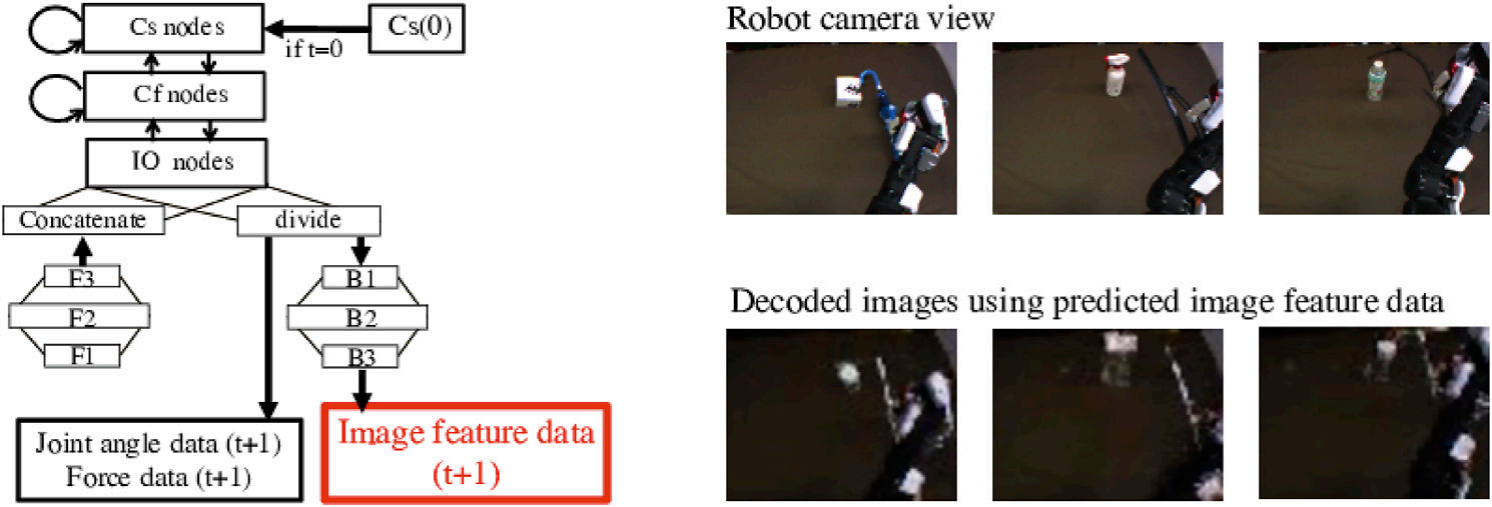
Decoded images using predicted image features output from the motion generation module. The original camera images are taken during the movement dealing with the unknown tools and the objects shown in the bottom of Figure 2. Although the decoded images shows similar color of the training tools and objects since the feature extraction module recognizes their features based on the training ones, they represent the shape, size, position, and orientation of the tools and objects in the robot camera view. They also show the position of the robot arm. It can be confirmed that the motion was generated while capturing the real time state during the movement.

Figure 10 shows the decoded images using predicted image feature data output by the motion generation module. The original camera images are taken during the Generalization Evaluation experiments. The decoded images represent the shape, size, position, and orientation of the tools and objects used. They also show the position of the robot arm. It can be confirmed that the motion was generated while capturing the real time state during the movement. Note that, the color and appearance of the tools or objects in the reconstructed images are close to the ones of training data because the feature extraction module recognizes the feature of the tool or object based on the training data.

## 6 Discussion

For summary, the tool-use model has two neural network modules: 1) a feature extraction module with a CAE trained to extract visual features from captured raw images, and 2) a motion generation module with a MTRNN and fully connected layers that integrates and predicts multimodal sensory-motor information. Through training of image reconstruction by the feature extraction module, the robot could extract image features from raw images captured by its camera. The motion generation module learns coordination of image feature data, joint angle data, and force sensor data, and performs next-step data predictions. In addition, the motion generation module can express the relationship of tools, objects, actions and effects with an internal latent space value, Cs(0). Using a provided goal image, the robot can generate actions by exploring Cs(0) values matched to the task.

Analyzing after training the DNN using task experience data, the tool-use model is able to self-organize tools, objects, and actions, and to automatically create maps representing their features in the Cs(0) space. With Cs(0) values, the tool-use model expresses the combination of the tools, objects, and actions needed to produce the target effects. In other words, the tool-use model understands combinations of the tools, objects, actions, and effects, not individually, meaning it acquires the relationships among the four factors. Then, we performed two experiments that confirmed the learning model’s accuracy and generalizability. The robot succeeded in 81% of tasks with unknown similar objects or tools. Moreover, the robot demonstrated task executions that reproduced target situations with unknown, complicated, totally different tools and objects. The results demonstrate that the features of tools and objects can be detected, and optimal actions can be generated based on the acquired relationships and the real time situations. In summary, the robot gained the relationships through its own experience, allowing it to consider combinations of tools, objects, and actions necessary to achieve its goal effects. This study is valuable as a novel robot control system. At first, the tool-use model does not require pre-calculation or pre-definition of tools and objects, nor motor command instructions from humans. We can assign tasks just by providing a goal image. Second, we can construct the tool-use model with relatively small training cost and make the model adaptable to unknown targets. Our work requires 200 training data for 10.7 s each. It took us only half a day to record that data. On the other hand, like methods using reinforcement learning require a large amount of training data, and it is difficult to record the data with actual robots. Studies that use simulation environments can overcome the difficulty of collecting data, however the difficulty of having to make up for the gaps from the real environment remains. In addition, our tool-use model shows broader generalization capabilities compared to previous studies. By acquiring the relationships among the four factors, our model has achieved what was not realized in many other research: it can handle both tools and objects which are unknown, and it can flexibly adjust actions in real time. Finally, multimodal learning solved the occlusion problem, and increased the range of objects that can be handled and the actions that can be taken. In the previous study Ref. Saito et al. (2018a), the adopted sensor was only image without force, so they could not use small objects and had many restrictions on the types of actions, because if an object was occluded by the arm, it would be misrecognized. Thanks to these achievement, our model can contribute to the realization of robots that can handle various tasks, it can greatly impact practical applications for robots in everyday environments. It is also useful for production at small quantities and wide variety.

However, there are some limitations in this work. First, tools and objects that change features like color, shape, or size during movement cannot be used, because it is difficult to explore Cs(0) values and detect the features if the goal image significantly differs from the initial image. Second, if objects or tools significantly differ from the trained ones, it will be difficult to handle them. If the appearances of them or effects are completely different, the feature extraction module and the motion generation module need to be trained again. Moreover, if their weights are too light or friction is too small, making them too easy to move. The robot would then struggle to operate them, because the motion generation model cannot predict next joint angles that largely differ from current ones, so there is a limit to the speed range that can be output.

In future studies, we will improve the tool-use model so that robots select tools suited to the situations and objects. In this model, the robot is initially grasping a tool, so while the robot considers how to move to accomplish its task, it does not consider tool selection. Reference Saito et al. (2018b) focused on only tool selection without considering relationships between objects, so we will combine these two studies so that robots must consider both how to move and which tool is best suited to accomplishing the task. Another area for future work is to have robots come up with novel ways to use tools. Realizing that ability would allow robots to use whatever tools happen to be available without instructions on their use, possibly generating unexpected behaviors.

## 7 Conclusion

We realized a robot that could acquire the relationships among tools, objects, actions and effects enabling tool-use, even for tools and objects being seen for the first time. The tool-use model learns sensorimotor coordination and the relationships among the four factors by training with data recorded during tool-use experiences. Unlike previous studies, our DNN model can simultaneously consider tools, objects, actions, and effects with no pre-definitions. Moreover, the robot can treat unknown tools and objects based on the relationships by generalizability of the tool-use model. Another advantage is that the robot can generate motions step-by-step, not just by choosing and replaying designed motor commands, so the robot can adjust its motions to cope with uncertain situations. Finally, the robot can find task goals and accomplish them just by providing goal images. This is one of the easiest ways to demonstrate to the robot the desired position and orientation. We confirmed the accuracy and generalization ability of the tool-use model with real robot experiments.

## Data Availability

The raw data supporting the conclusions of this article will be made available by the authors, without undue reservation.
